# Clinical Outcomes of Right Ventricular Apical, Septal, and Conduction System Pacing in the Medicare Population

**DOI:** 10.1161/CIRCEP.125.013940

**Published:** 2025-08-22

**Authors:** Pugazhendhi Vijayaraman, Colleen Longacre, Jordana Kron, George H. Crossley

**Affiliations:** 1 Division of Electrophysiology, Geisinger Heart Institute, Wilkes-Barre, PA (P.V.).; 2 Medtronic Inc., Mounds View, MN (C.L.).; 3 Division of Electrophysiology, Virginia Commonwealth University, Richmond (J.K.).; 4 Division of Electrophysiology, Vanderbilt Univ Medical Center, Nashville, TN (G.H.C.).

**Keywords:** heart failure, hospitalization, Medicare, mortality, pacemaker, artificial

Conduction system pacing (CSP) has emerged as an alternative to right ventricular (RV) pacing and has demonstrated clinical benefits for patients with bradycardia in both clinical studies^1^ and real-world cohorts.^2^ However, few studies have further disaggregated RV pacing into RV septal pacing (RVSP) versus RV apical pacing (RVAP) and have mostly focused on acute implant metrics such as QRS duration.^3^ Few have compared long-term benefits across these 3 pacing modalities. This analysis aimed to compare CSP versus RVSP versus RVAP in a large, population-based cohort of US Medicare beneficiaries. The objectives were to compare rates of incident heart failure hospitalization (iHFH) and a composite end point of iHFH+all-cause mortality (ACM) at 12 months.

This study utilized data from the Micra CED study (Micra Coverage With Evidence Development),^4^ a continuously enrolling, longitudinal cohort study that follows all Medicare patients implanted with either a leadless or transvenous pacemaker. Medicare administrative claims data were used to identify patients implanted with either a single-chamber or dual-chamber transvenous pacemaker from 2020 to 2022.

Lead placement data from Medtronic’s device registration system were used to identify patients treated with CSP using a 3830 catheter-delivered lead or RVSP and RVAP delivered via a 4076/5076 lead at the same centers using previously described methodology.^2^ Patient characteristics, comorbidities, and outcome measures were ascertained from the Medicare claims data using the relevant diagnostic and procedure codes. An iHFH was defined as a hospitalization with an *International Classification of Diseases*, *Tenth Revision* diagnosis code for heart failure in the primary position on an inpatient claim after discharge from the implant procedure hospitalization or encounter in a patient with no evidence of heart failure at baseline. iHFH can provide evidence of and serve as a proxy measure for pacing-induced cardiomyopathy or potentially worsening heart failure among pacemaker patients.^4^ Cause of death cannot be determined from the Medicare data; therefore, only ACM is reported. Medicare claims data were available through December 31, 2022; patients without an event were censored on that date.

Cox proportional hazards models were used to compare iHFH and an iHFH+ACM composite end point at 12 months postimplant. Models were adjusted for patient demographics, comorbidities, pacemaker type (single- versus dual-chamber), and implant encounter characteristics. Standard errors were correlated at the hospital level to account for within-hospital correlation. Statistical significance was set at *P*<0.05. The study was approved by Western-Copernicus Group Institutional Review Board with a waiver of informed consent; data access is restricted by the Medicare data use agreement.

A total of 6256 patients treated with CSP, 17 565 treated with RVSP, and 15 418 patients treated with RVAP pacing were included in our study cohort (Figure [A]). Over the 3-year study period, CSP placement increased as a percentage of total lead placement mix from 10% in 2020% to 22% in 2022, while RV apical lead placement decreased from 46% in 2020% to 32% in 2022. RV septal lead placement remained relatively stable: 43% in 2020 and 45% in 2022. Patients with CSP had statistically significantly lower composite iHFH+ACM compared with both patients with RVSP (adj HR [adjusted hazard ratio], 0.73 [95% CI, 0.65–0.83]; *P*<0.0001) and patients with RVAP (adj HR, 0.70 [95% CI, 0.62–0.79]; *P*<0.0001). The difference in iHFH+ACM between RVSP and RVAP was not statistically significant (RVSP: adj HR, 0.95 [95% CI, 0.88–1.03]; *P*=0.24; Figure [B]).

**Figure.**
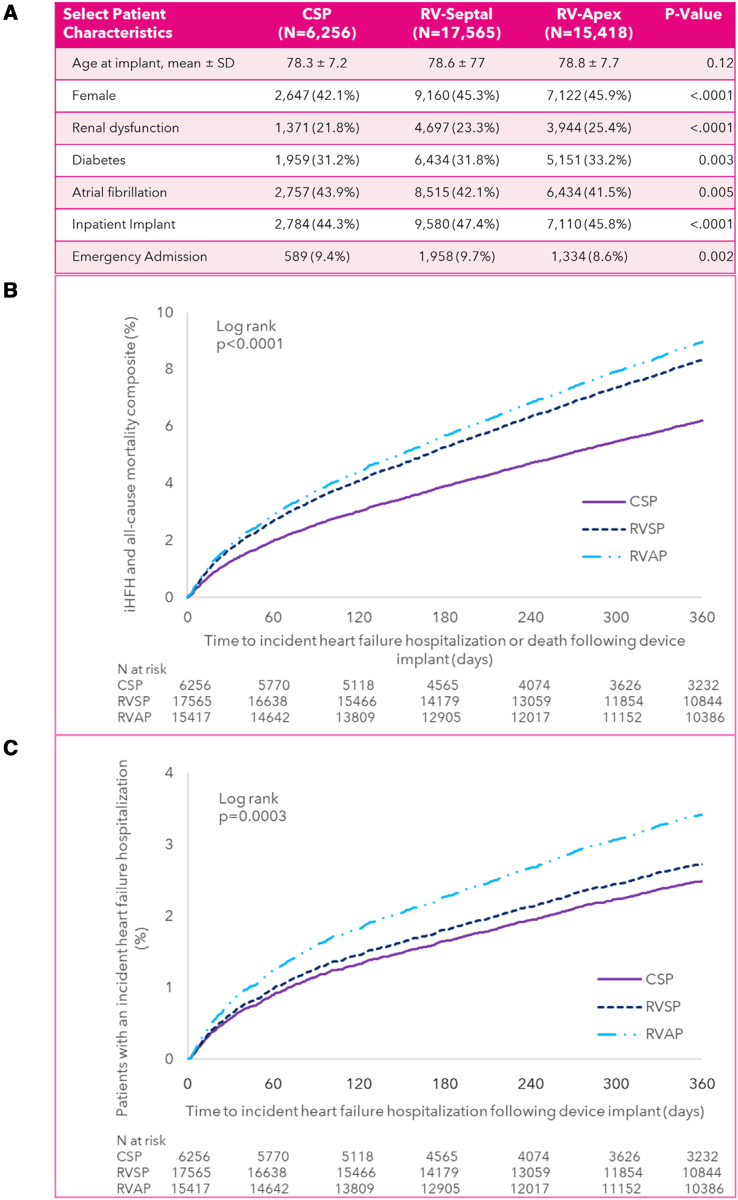
**Baseline characteristics and clinical outcome.** Select patient cohort characteristics (**A**), adjusted time to event plots for incident heart failure hospitalization (iHFH)+all-cause mortality composite end point (**B**), and iHFH (**C**) over 12 months in patients treated with conduction system pacing (CSP) vs right ventricular septal pacing (RVSP) vs right ventricular apical pacing (RVAP).

However, compared with patients treated with RVAP, both patients treated with CSP as well as patients treated with RVSP had statistically significantly lower iHFH at 12 months (CSP: adj HR, 0.73 [95% CI, 0.60–0.89]; *P*=0.002 and RVSP: adj HR, 0.81 [95% CI, 0.71–0.92]; *P*=0.002). The difference in iHFH between CSP and RVSP was not statistically significant (adj HR, 0.91 [95% CI, 0.74–1.11]; *P*=0.33; Figure [C]).

This analysis aligns with prior studies demonstrating the clinical benefits associated with CSP versus traditional RVP with respect to reduced risk of HFH and mortality.^5^ It further provides evidence to suggest that within RVP, RVSP may reduce some of the risk associated with RVAP, as iHFH were significantly lower with RVSP versus RVAP in our study cohort. This study has several limitations, including its observational research design, which could lead to residual confounding due to selection bias across study groups not entirely overcome by statistical adjustment. In addition, we relied on lead placement as reported in the manufacturer device registration database; we cannot confirm via imaging or ECG. Finally, we cannot report on device metrics such as pacing thresholds, pacing percentage, or QRS duration.

In our analysis of nearly 40 000 Medicare patients, CSP was associated with a greater reduction in the combined iHFH+ACM end point at 12 months compared with RVAP or RVSP, and both CSP and RVSP were associated with reduced iHFH compared with RVAP. Though observational, our results suggest the benefits of physiological pacing may accrue incrementally as lead placement moves from apical to septal to CSP location.

## ARTICLE INFORMATION

### Sources of Funding

This study was supported by Medtronic Inc.

### Disclosures

Dr Vijayaraman has received honoraria and research support from and is a consultant and serves on the advisory board for Medtronic, serves on the advisory board of and is a consultant for Abbott, and has received honoraria from Biotronik and Boston Scientific. He owns a patent for His bundle pacing delivery tool. Dr Crossley is supported by Clinical and Translational Science Award award no. UL1 TR002243 from the National Center for Advancing Translational Sciences and consults for Medtronic, Boston Scientific, and is on the Speakers Bureau for Medtronic and Spectranetics. Dr Longacre is an employee and shareholder of Medtronic. The other author reports no conflicts.
